# Involvement of Global Genome Repair, Transcription Coupled Repair, and Chromatin Remodeling in UV DNA Damage Response Changes during Development

**DOI:** 10.1371/journal.pgen.1000941

**Published:** 2010-05-06

**Authors:** Hannes Lans, Jurgen A. Marteijn, Björn Schumacher, Jan H. J. Hoeijmakers, Gert Jansen, Wim Vermeulen

**Affiliations:** 1 Department of Genetics, Medical Genetics Center, Erasmus MC, Rotterdam, The Netherlands; 2 Cologne Excellence Cluster for Cellular Stress Responses in Aging-Associated Diseases (CECAD), Cologne, Germany; 3 Department of Cell Biology, Medical Genetics Center, Erasmus MC, Rotterdam, The Netherlands; University of Washington, United States of America

## Abstract

Nucleotide Excision Repair (NER), which removes a variety of helix-distorting lesions from DNA, is initiated by two distinct DNA damage-sensing mechanisms. Transcription Coupled Repair (TCR) removes damage from the active strand of transcribed genes and depends on the SWI/SNF family protein CSB. Global Genome Repair (GGR) removes damage present elsewhere in the genome and depends on damage recognition by the XPC/RAD23/Centrin2 complex. Currently, it is not well understood to what extent both pathways contribute to genome maintenance and cell survival in a developing organism exposed to UV light. Here, we show that eukaryotic NER, initiated by two distinct subpathways, is well conserved in the nematode *Caenorhabditis elegans*. In *C. elegans*, involvement of TCR and GGR in the UV-induced DNA damage response changes during development. In germ cells and early embryos, we find that GGR is the major pathway contributing to normal development and survival after UV irradiation, whereas in later developmental stages TCR is predominantly engaged. Furthermore, we identify four ISWI/Cohesin and four SWI/SNF family chromatin remodeling factors that are implicated in the UV damage response in a developmental stage dependent manner. These *in vivo* studies strongly suggest that involvement of different repair pathways and chromatin remodeling proteins in UV-induced DNA repair depends on developmental stage of cells.

## Author Summary

Nucleotide Excision Repair (NER) removes many forms of helix-distorting DNA damage which interfere with transcription and replication, including those induced by UV irradiation. NER is initiated when damage is sensed during transcription, i.e. Transcription-Coupled Repair (TCR), or when damage is sensed in non-transcribed genomic sequences, i.e. Global Genome Repair (GGR). Although the molecular mechanism of the core NER is known, it is not well understood how the UV response functions in living organisms and which additional mechanisms are involved to regulate its efficiency. Therefore, we exploited the small soil nematode *C. elegans* to study the UV response in a living organism. Using different NER–deficient animals, we found that in early development mainly GGR, but in later development mainly TCR is active in the UV response. Furthermore, we identified several new chromatin remodeling factors, whose involvement in the UV response also differs during development and which are thought to regulate efficiency of the UV response by altering chromatin structure. Our studies show that *C. elegans* is very well suited to genetically analyze the UV response during different developmental stages and in different tissues in a living animal.

## Introduction

A network of DNA damage response (DDR) mechanisms protects organisms against the continuous genotoxic stress induced by reactive metabolites and other genotoxic agents, such as environmental contaminants and ultraviolet (UV) radiation from the sun [1]. The DDR network consists of several DNA repair mechanisms, cell cycle checkpoints and cellular senescence and apoptotic signaling cascades. Nucleotide Excision Repair (NER) is a DNA repair mechanism that is able to remove a wide variety of helix-destabilizing DNA lesions including those induced by UV light.

Eukaryotic NER is a highly conserved multi-step process, involving more than 25 proteins, of which the principal molecular mechanism has been dissected in detail [1], [2]. NER is initiated by two distinct DNA damage recognition mechanisms which use the same machinery to repair the damage. Damage in the transcribed strand of active genes is repaired by Transcription Coupled Repair (TCR), which depends on recruitment of the ATP-dependent chromatin remodeling protein Cockayne Syndrome protein B (CSB) and the WD40 domain containing protein Cockayne Syndrome protein A (CSA) to the site of damage [3]–[5]. TCR is thought to be activated by stalling of elongating RNA polymerase II during transcription [3], [6]. Damage in other, non-transcribed sequences of the genome is repaired by Global Genome Repair (GGR), which requires detection of the lesions by the UV-damaged DNA-binding protein (UV-DDB) complex and a complex containing Xeroderma Pigmentosum group C protein (XPC), human homolog of RAD23 (hHR23) and Centrin-2 [7]–[9]. The XPC protein is essential for the initiation of GGR and subsequent recruitment of other NER factors [10], [11]. The majority of XPC is found in complex with the hHR23B protein, while only a fraction copurifies with the redundant hHR23A protein. Both hHR23 proteins are thought to stabilize XPC and stimulate its function [12]–[14]. Although HR23B is not essential for *in vitro* NER, *in vivo* damage is poorly repaired in cells lacking hHR23B [12], indicating that hHR23B is essential for proper NER function. Following detection of a lesion, either via GGR or TCR, the transcription factor IIH (TFIIH) is recruited to open the DNA helix around the damage in an ATP-dependent manner using its Xeroderma Pigmentosum group B and D (XPB and XPD) helicase subunits [1], [2]. Next, Xeroderma Pigmentosum group A (XPA) and Replication Protein A (RPA) are recruited to stabilize the repair complex and properly orient the structure-specific endonucleases Xeroderma Pigmentosum group F (XPF)/Excision Repair Cross-Complementing protein 1 (ERCC1) and Xeroderma Pigmentosum group G (XPG) to excise the damaged strand. The resulting ∼30 nt single strand DNA gap is filled by DNA synthesis and ligation.

In mammals, congenital defects in GGR and TCR lead to an increased sensitivity towards DNA damaging agents such as UV irradiation. Inherited mutations in GGR genes cause Xeroderma Pigmentosum, which is characterized by extreme UV-sensitivity and skin cancer predisposition [15]. Hereditary TCR deficiency causes Cockayne syndrome, which leads to entirely different features such as severe but variable neurodevelopmental symptoms and premature aging. In contrast to mammals, specific TCR defects in yeast have only a marginal effect on DNA damage resistance, despite a relatively larger proportion of the genome that is transcriptionally active [16].

Current knowledge of NER does not provide an explanation for the pleiotropic phenotypic expression of NER-deficiencies. Despite detailed insight in the molecular mechanism of NER, many aspects of the *in vivo* UV-induced DNA damage response (UV-DDR) are still unclear. It is for instance not well understood how NER functions in nucleosomal DNA and in different tissues of developing organisms. Therefore, a full understanding of the complete UV-DDR and its interplay with NER in living organisms is imperative. The nematode *C. elegans* seems well suited to analyze the complete UV-DDR *in vivo* in more detail, because of its short lifetime, well-characterized biology and its amenable use to identify new genes involved in the UV-DDR. Several studies have specifically addressed the role of NER proteins in the UV-DDR in *C. elegans*. Knockdown of the *C. elegans* orthologs of mammalian CSB, XPA and XPF increases sensitivity to UV irradiation [17]–[21]. Furthermore, it was shown that the XPA and XPC orthologs function in the *C. elegans* germ line to induce cell cycle arrest and apoptosis in response to UV irradiation [22]. Together, these studies suggest that NER function is highly conserved in *C. elegans*. However, a thorough analysis of the function of NER and, more specifically, the role of the GGR and TCR subpathways in response to UV irradiation in different tissues during development has not been performed.

In this study, we make use of mutations in the *C. elegans* RAD23, XPC and CSB orthologs to show that during early development, in germ cells and embryos, GGR is the major pathway involved in the response to UV irradiation. Defective GGR leads to inefficient lesion removal in germ cells, specific defects in germ cell development and embryonic death after UV irradiation. Intriguingly, in juvenile and adult animals TCR is the major NER pathway involved in the UV response. Analysis of the UV response of embryos shows that, during development, TCR gradually becomes more important than GGR. Finally, we exploit *C. elegans* to identify novel genes involved in the UV-DDR, specifically in the TCR-related UV response. Our results reveal four genes implicated in SWI/SNF and four genes implicated in ISWI ATP-dependent chromatin remodeling whose involvement in the UV-DDR changes during development.

## Results

### General NER–deficient and GGR– and TCR–specific mutants

**Figure 1:**
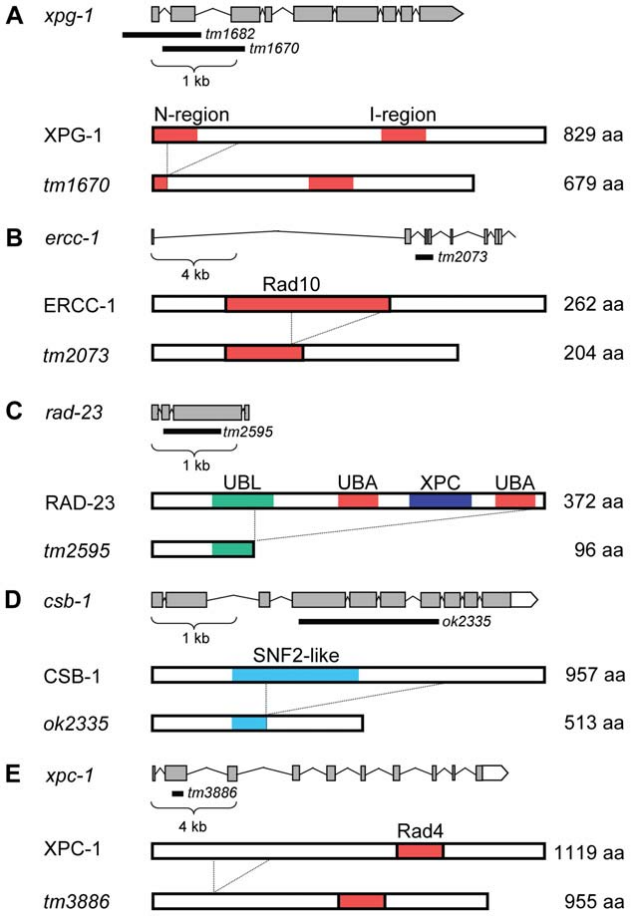
Representations of wild-type and mutant *C. elegans* XPG-1, ERCC-1, RAD-23, CSB-1, and XPC-1. Representations of the genomic (top) and protein (bottom) organization of each gene/protein are depicted. (A) The typical N-terminal and Internal catalytic sites of XPG-1 are depicted. *tm1670* represents a deletion that is predicted to remove amino acids 31–80, deleting part of the N-terminal catalytic domain. (B) The conserved Rad10 domain is predicted to be partially deleted by the *tm2073* deletion of *ercc-1*. (C) Wild type *C. elegans* RAD-23 protein contains four domains that are also found in human RAD23 proteins: one N-terminal UBiquitin Like (UBL), two UBiquitin Associated (UBA) domains and an XPC binding domain. The *tm2595* allele deletes most of the conserved domains. (D) CSB-1 protein contains one SWI/SNF domain, of which the large part is removed by the *ok2335* deletion. (E) *tm3886* represents a deletion that is predicted to create a truncated XPC-1 protein. Mutant protein predictions were according to FGENESH.

To study the UV-DDR in the context of a whole organism, we tested UV-B sensitivity of mutant *C. elegans* at different developmental stages. Our initial experiments showed that UV-B irradiation produced better reproducible phenotypes than UV-C irradiation (data not shown), most likely due to the fact that UV-B penetrates deeper through the multiple cell layers of *C. elegans*. First, we tested UV sensitivity of animals carrying mutations in the general NER genes *xpa-1*, *xpg-1*, *xpf-1* and *ercc-1*. Alleles of *xpa-1* and *xpf-1*, but not *xpg-1* and *ercc-1*, have been previously described. *xpa-1(ok698)* encodes a putative null allele of the *C. elegans* ortholog of mammalian XPA and was shown to cause severe sensitivity to UV irradiation [20], [22]. *him-9(e1487)* is an allele of *xpf-1*, encoding the *C. elegans* ortholog of mammalian endonuclease XPF [23]. *tm1682* and *tm1670* are two alleles of *xpg-1*, the ortholog of the mammalian endonuclease XPG and have not been described before. *tm1682* represents a deletion of the first two exons of *xpg-1*, probably creating a knock-out allele, but also of part of the last exon of the adjacent glycosyl hydrolase gene *tre-1* (Figure 1A). Thus, to rule out an effect of *tre-1*, in our analysis we also included *tm1670*, which represents a deletion that is predicted to remove exon 2 and a large part of exon 3, encoding for a truncated 679 amino acids in stead of 829 amino acids protein (Figure 1A). Since most of the N-terminal nuclease domain is deleted, the resulting protein is expected to be non-functional. *tm2073* represents a deletion in the conserved Rad10 domain of *ercc-1*, the *C. elegans* ortholog of mammalian ERCC1 which is in complex with XPF, and is predicted to encode a loss-of-function allele (Figure 1B).

To address the specific contribution of the TCR and GGR pathways in the UV-induced DDR *in vivo*, we also analyzed *C. elegans* strains carrying mutations expected to affect either pathway specifically. The genome of *C. elegans* encodes an ortholog of the GGR-specific mammalian HR23A and HR23B genes, called *rad-23*. This gene is predicted to encode for a 372 amino acids protein having similar domain organization as mammalian HR23A and HR23B proteins (Figure 1C; [24]). The *rad-23(tm2595)* allele represents a deletion of the major parts of exon 2 and exon 3 and an insertion of 28 basepairs. Since *tm2595* deletes both UBA domains and the XPC binding domain and is predicted to encode a truncated protein of 96 aa, this allele is likely a functional null allele.

The TCR-specific mammalian CSB gene is represented in *C. elegans* by *csb-1* [17]. This gene encodes a 957 amino acids protein containing a SNF2-like ATPase domain, similar to human CSB (Figure 1D). The *csb-1(ok2335)* allele consists of a 1620 bp deletion which removes exon 5 and 6 and the largest parts of exon 4 and 7. This allele is predicted to encode a truncated protein of 513 amino acids, which is likely a functional null since most of the SNF2 domain is deleted.

### GGR but not TCR is essential for survival of UV-irradiated germ cells

**Figure 2:**
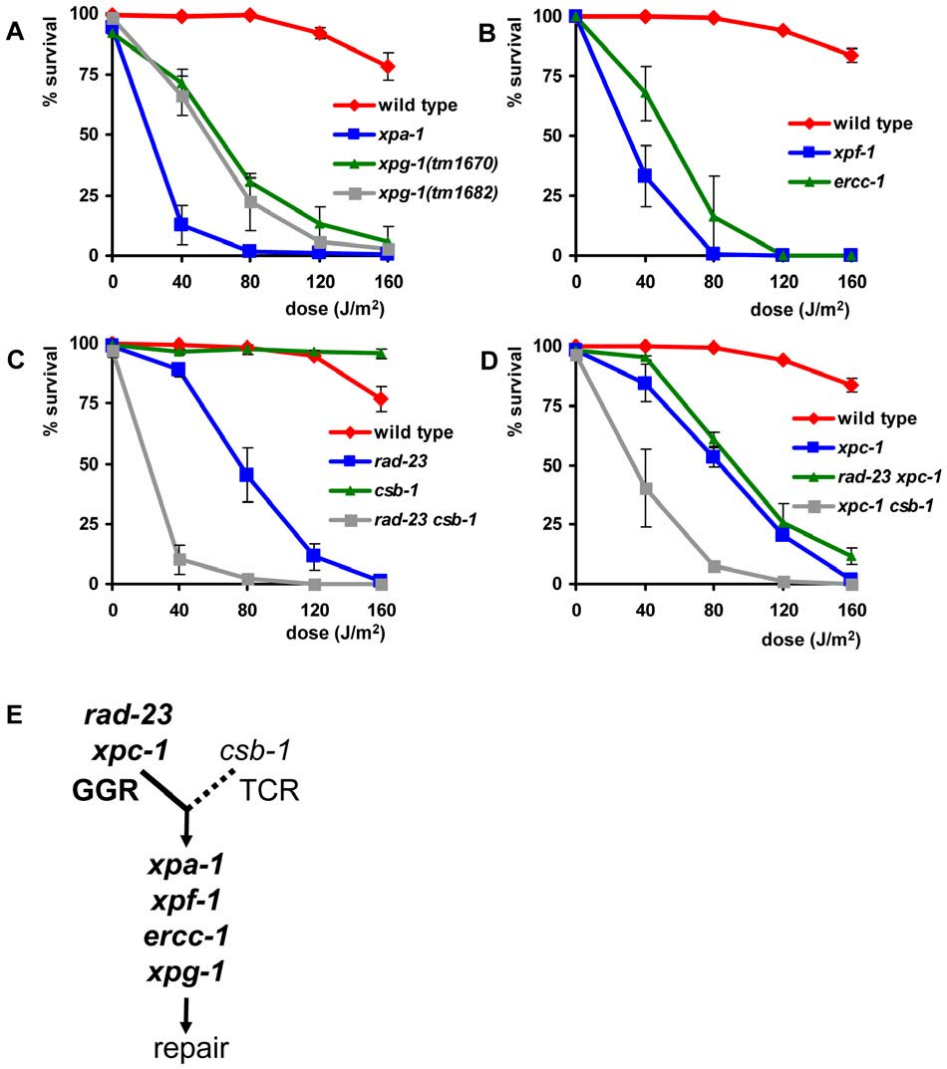
Germ cell and embryo survival following UV irradiation. The percentages (survival) of hatched eggs following UVB irradiation are plotted against the indicated applied UVB-doses on germ cells in young adult animals carrying mutations in general NER factors (A and B), in *rad-23* and *csb-1* single and double mutants (C) and in *xpc-1* single and *rad-23; xpc-1* and *xpc-1; csb-1* double mutants (D). (E) shows a simplified model of GGR and TCR in the germ line of *C. elegans*. Data for *xpf-1* and *ercc-1* in (B) were normalized because these mutants produce ∼20–25% unviable eggs without UV irradiation. Each line represents the mean of two or more independent experiments (typically, n>40 eggs). However, for *xpa-1*, *xpg-1*, *xpf-1*, *ercc-1*, *rad-23; csb-1* and *xpc-1; csb-1* mutants high UV doses severely decreased the amount of eggs laid. Survival was scored as zero if no eggs were laid. Error bars denote the s.e.m.

To test UV sensitivity of germ cells, adult animals were irradiated and allowed to recover for 24 hours, after which they were put on fresh plates to lay eggs for 3–4 hours (Figure S1A). ‘Germ cell and embryo survival’ was measured by determining the percentage of eggs that hatched over the total amount of eggs laid. As expected, we found that the core NER factors *xpa-1*, *xpg-1*, *xpf-1* and *ercc-1* were necessary for germ cells and embryos to survive even relatively low doses of UV irradiation (Figure 2A and 2B).

Next, we tested UV sensitivity of *rad-23(tm2595)* and *csb-1(ok2335)* mutants. Functional *rad-23* appeared to contribute only partially to UV resistance (compared to *xpa-1*), whereas, surprisingly, *csb-1* did not seem to contribute at all (Figure 2C). Similar results were obtained using eggs laid immediately after irradiation or after different recovery periods up to 51 hrs after irradiation (data not shown). This suggests that a similar UV response, involving general NER factors and *rad-23*, but not *csb-1*, acts in all developing germ cells, oocytes and early embryos.

The specific contribution of *rad-23* but not *csb-1* suggests that germ and early embryonic cells depend mainly on the GGR pathway of NER to overcome the effects of UV irradiation. Alternatively, it could be that *csb-1* is not involved in TCR in *C. elegans* or that TCR defects are not associated with UV sensitivity. To test whether GGR and TCR act redundantly in the germ line, or whether *csb-1* is not involved in UV-damage repair or survival, we generated animals carrying mutations in both *rad-23* and *csb-1*. Irradiation of *rad-23; csb-1* double mutants showed that these animals are more UV sensitive than *rad-23* single mutants and as sensitive as animals carrying mutations in general NER genes (Figure 2C). This suggests that both TCR and GGR are active in germ cells.

In mammals, RAD23 functions in GGR as part of a heterotrimeric complex containing also Centrin-2 [7], [25] and XPC [8], [26]. The genome of *C. elegans* contains an ortholog of XPC, *xpc-1*, for which only recently, during the course of our experiments, a good loss-of-function allele became available. This allele, *tm3886*, represents a 24 bp insertion and 474 bp deletion in exon 2, probably causing a truncated protein (Figure 1E). To confirm that the specific UV sensitivity of *rad-23* is caused by a defect in GGR, we tested the phenotype of the novel *xpc-1* mutation. *xpc-1* single mutants showed a similar UV sensitivity in the germ line as *rad-23* single and *rad-23; xpc-1* double mutants, whereas *xpc-1; csb-1* double mutants were as UV sensitive as *rad-23; csb-1* double mutants (Figure 2D). These results are in line with our previous findings and with the idea that in *C. elegans*, similar as in mammals, RAD-23 and XPC-1 function in complex during GGR.

Based on our results (summarized in Table 1), we hypothesize that in the germ line GGR plays an essential role in UV survival, whereas TCR only has a secondary, partially redundant function to GGR (Figure 2E). Furthermore, our results are in agreement with the idea that, similar as in mammals, *rad-23/xpc-1* and *csb-1* act in parallel pathways, GGR and TCR, that converge on a common pathway to repair DNA damage.

**Table 1:** Overview of UV sensitivity of different mutants.

*C. elegans*	mammalian	UV sensitivity	
genotype	ortholog	germ cell	L1 larvae
wild type		−	−
*xpa-1*	XPA	+	+
*xpg-1*	XPG	+	+
*xpf-1*	XPF	+	+
*ercc-1*	ERCC1	+	+
*rad-23*	HR23B	+	−
*csb-1*	CBS	−	+
*xpc-1*	XPC	+	−
*rad-23; csb-1*		+	+
*rad-23; xpc-1*		+	−
*xpc-1; csb-1*		+	+
*isw-1*	ISWI/SMARCA5	+	+
*him-1*	SMC1	+	+
*hda-2*	HDAC1	−	+
*hda-3*	HDAC1	−	+
*psa-4*	BRM/SMARCA2	+	+
*pbrm-1*	PBRM1	−	+
*snfc-5*	SMARCB1	−	+
*psa-1*	SMARCC1	−	+

Summarized results of Figure 2, Figure 5, Figure 6, and Figure 7. For each gene UV sensitivity in the germ cell and embryo or in the L1 larvae assays is show. + denotes UV hypersensitive, − denotes not UV hypersensitive, compared to wild type.

### GGR maintains germ cells in response to UV irradiation

**Figure 3:**
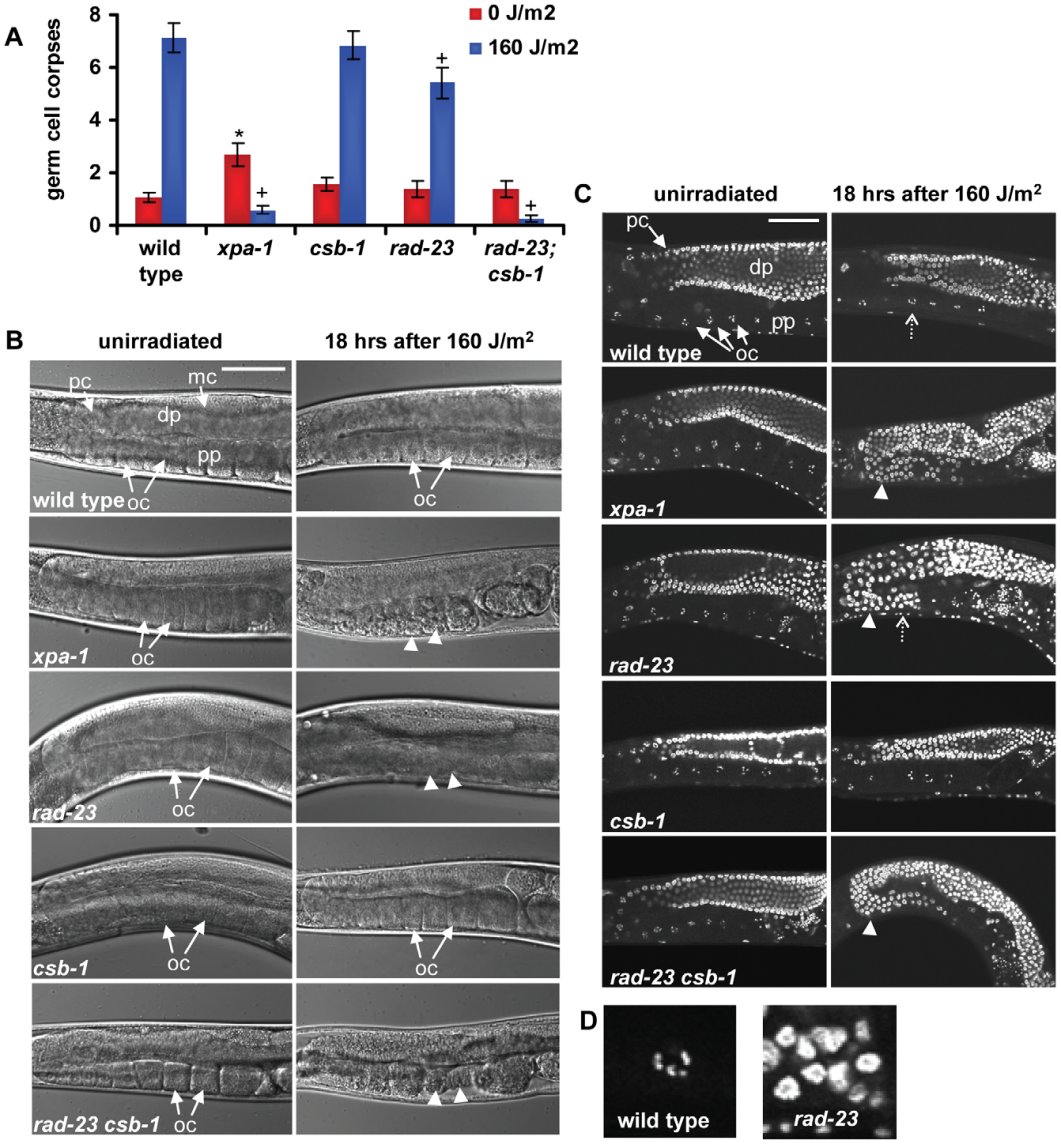
Germ cell responses to UVB irradiation. (A) Amount of apoptotic cells 6 hours following no or 160 J/m^2^ UVB irradiation is shown. Each bar represents two independent experiments. Error bars denote s.e.m. Significant differences (p<0.05) compared to wild type (*) and 160 J/m^2^ irradiated wild type (+) are indicated. (B) Nomarski images of unirradiated and irradiated gonads, 18 hrs after 160 J/m^2^ UVB. In *xpa-1*, *rad-23*, and *rad-23; csb-1* mutants, the appearance of the proximal gonad part drastically changes over time after irradiation. In the first image, mitotic cells (mc), pachytene cells (pc), oocytes (oc, arrows), the distal part (dp) and the proximal part (pp) of the gonad are indicated. Arrowheads indicate morphological changes in oocytes after UV. Bar in first image represents 50 µm. (C) Germ cells of animals 18 hrs after no or 160 J/m^2^ UVB irradiation, fixed and stained with DAPI. In irradiated *xpa-1*, *rad-23* and *rad-23; csb-1* animals, pachytene like germ cells progress beyond the gonad bend (indicated by arrowheads) in contrast to wild type and *csb-1* animals. In the first image, pachytene cells (pc), diakinesis oocytes (oc, arrows), the distal part (dp) and the proximal part (pp) of the gonad are indicated. Typical oocytes are easily distinguishable due to their condensed homologous chromosomes pairs. Bar in first image represents 50 µm. The areas indicated by the dashed open arrows for irradiated wild type and *rad-23* are enlarged in (D).

Previously, it was found that ionizing and UV irradiation both induce apoptosis of a fraction of the pachytene germ cells of *C. elegans* [22], [27], which are located near the gonad tube bend (Figure 3B, first image). Functional *xpa-1* was shown to be required for induction of apoptosis [22], [27], suggesting that the NER process itself is necessary to activate the apoptotic machinery. To test whether induction of germ cell apoptosis requires functional GGR or TCR, we measured induction of apoptosis in the pachytene germ cells of wild type, *xpa-1*, *rad-23*, *csb-1* and *rad-23;csb-1* mutants in response to UVB irradiation. In contrast to wild type animals, *xpa-1* mutants exhibited severely reduced apoptosis induction after UVB, as observed previously (Figure 3A; [22]). Furthermore, we found that in *csb-1* mutants apoptosis was induced at wild type levels, whereas in *rad-23* mutants apoptosis induction was mildly reduced. No apoptosis induction after UV irradiation was observed in *rad-23; csb-1* double mutants, similar as in *xpa-1* mutants. These results indicate that both the GGR and the TCR pathway are required to induce germ cell apoptosis in response to UV. Together with the mild decrease in apoptosis induction in *rad-23* mutants, this is in line with our previous results showing that GGR, acting partially redundant with TCR, is the main NER pathway in the germ line of *C. elegans*.

Surprisingly, UV irradiation does not induce, but even seems to inhibit apoptosis in *xpa-1* and *rad-23; csb-1* mutants, and less efficiently in *rad-23* mutants. In unirradiated animals, germ cell apoptosis is thought to be a developmental mechanism to maintain germ line homeostasis [28]. Following UV irradiation, NER-dependent apoptosis of pachytene stage germ cells may serve to eliminate damaged cells. After exiting pachytene stage, undamaged germ line nuclei progress to complete meiosis and are fertilized as oocytes in the proximal part of the gonad (reviewed in [29]; Figure 3B, first image). Next, fertilized oocytes initiate embryogenesis. Thus, it was interesting to follow the fate of UV-damaged pachytene germ cells in NER proficient and deficient animals. In wild type and *csb-1* animals, oocytes in the proximal part of the gonad appeared morphologically normal after UV irradiation. In contrast, in *xpa-1*, *rad-23* and *rad-23; csb-1* mutants, the morphology of oocytes was drastically altered over time after UV irradiation (Figure 3B, arrowheads). Further analysis using DAPI staining to visualize chromatin condensation associated with specific meiotic developmental stages revealed that in *xpa-1*, *rad-23* and *rad-23; csb-1* mutant germ cells failed to progress to the oocyte stage for at least up to 30 hrs after irradiation (Figure 3C, arrowheads, and Figure 3D; data not shown). In contrast, in wild type animals and *csb-1* mutants morphologically normal diakinesis stage oocytes were readily recognizable at all time points after UV irradiation (Figure 3C and 3D and data not shown). These results suggest that in UV irradiated animals lacking functional XPA-1 or RAD-23 maturation of meiotic germ nuclei is impaired. Further down the gonad tube, the general morphology of embryos *in utero* was also severely compromised in *xpa-1*, *rad-23* and *rad-23; csb-1* mutants (data not shown), suggesting extensive embryonic cell death. This latter finding is in agreement with the fact that fewer eggs are laid with increasing dosages of UV irradiation (data not shown; [30]) and that eggs which are laid show increased mortality rates. Possibly, the lack of UV-induced apoptosis in these mutants leads to a reduced clearance of UV-damaged cells which results in defects in meiotic maturation, morphological changes and ultimately cell death. Together, these results confirm that in germ cells GGR, but not TCR, is the dominant NER pathway necessary to overcome the genotoxic effects of UV irradiation.

**Figure 4:**
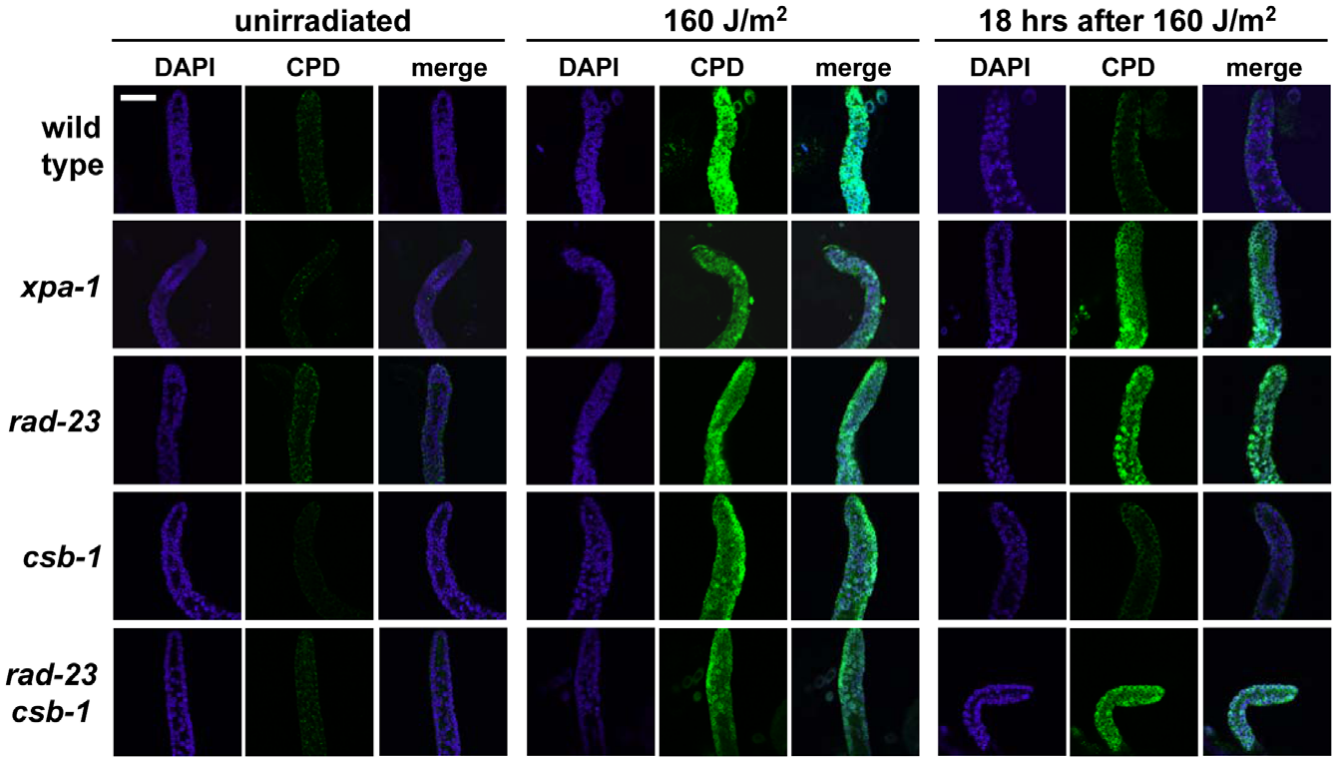
CPD repair in *C. elegans* germ cells. Immunofluorescence on dissected gonads shows that after 160 J/m^2^ UVB irradiation, CPD damage (green) is repaired in wild type and *csb-1* animals, but not in *xpa-1*, *rad-23* and *rad-23; csb-1* animals. Repair was visualized 18 hours after irradiation, as at this time point the majority of CPDs in *C. elegans* are repaired [52], [65], while the proliferation arrest caused by UV irradiation in distal mitotic germ cells still lasts [22]. To visualize germ cell nuclei, DAPI staining (blue) was used. Bar in first image represents 20 µm.

To investigate whether the UV hypersensitivity of germ cells of *xpa-1* and *rad-23* mutants is accompanied or caused by reduced DNA repair, we measured UV damage removal. To this end we applied immunofluorescence to visualize Cyclobutane Pyrimidine Dimers (CPDs), the most abundant UVB-induced DNA lesions [31]. As shown in Figure 4, 18 hours after irradiation a virtual complete removal of CPDs from gonad nuclei was observed in wild type and *csb-1* animals, but not in *xpa-1*, *rad-23* and *rad-23; csb-1* mutants. These results further corroborate the notion that GGR is the major NER pathway in germ cells of *C. elegans*.

### TCR, but not GGR, is essential for UV survival of larvae

**Figure 5:**
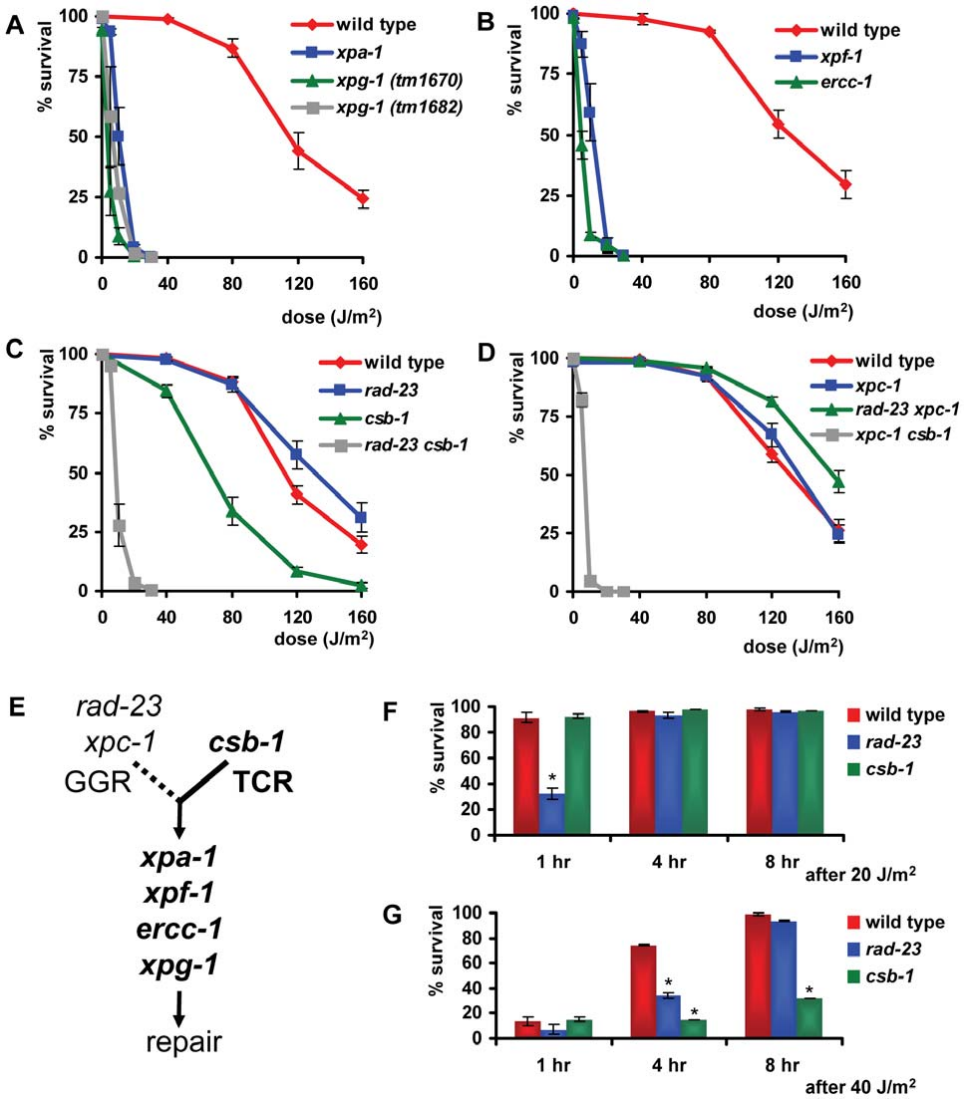
L1 larvae and egg survival following UV irradiation. The percentages (survival) of animals that developed beyond the L2 larval stage following UV irradiation are plotted against the indicated UVB-doses of L1 larvae (A–D) or eggs (F, G). (A, B) shows the survival of animals carrying mutations in general NER factors, (C) shows the survival of *rad-23* and *csb-1* single and double mutants, (D) shows the survival of *xpc-1* single and double mutants. (E) shows a simplified model of GGR and TCR in L1 larvae. In (F, G), eggs of wild type, *rad-23* and *csb-1* mutants were irradiated (20 and 40 J/m^2^, respectively) 1, 4 or 8 hours after collection using hypochlorite. In (A–D), each line represents the mean of two or more independent experiments, each performed in duplicate (typically, n>80). (F, G) show the mean of a representative experiment performed in duplicate (n>50 eggs). Significant differences (p<0.05) compared to wild type are indicated by an asterisk. Error bars denote the s.e.m.

To investigate whether the observed GGR dependence of the UV response is restricted to germ cells or whether it is a common feature of *C. elegans* cells, we determined UV sensitivity of later developmental stages. We found that early developmental stages of *C. elegans* are more sensitive to UV irradiation than later stages, in line with what was previously described (data not shown; [21], [30]). To score UV sensitivity of L1 larvae, we developed an assay in which survival of UVB-irradiated L1 larvae was measured (see materials and methods and Figure S1B). Survival was scored by determining the percentage of animals capable of growing to adulthood over the total amount of animals in response to UV irradiation. We found that *xpa-1*, *xpg-1*, *xpf-1* and *ercc-1* L1 larvae were extremely sensitive to UV and arrested development completely in response to relatively low UV doses (Figure 5A and 5B). This developmental arrest is possibly caused by a damage-induced block in transcription, causing breakdown of RNA polymerase II, as was shown following UVC irradiation of *xpa-1* mutants [20]. However, at the UVB doses we used (up to 160 J/m^2^) we were unable to confirm breakdown of RNA polymerase II (data not shown). To our surprise, we found that *csb-1* L1 larvae, but not *rad-23* L1 larvae, were more sensitive to UV than wild type animals (Figure 5C), opposite to what was observed in germ cells. Similar to *rad-23* mutant germ cells, *csb-1* L1 larvae showed an intermediate UV sensitivity in between wild type animals and general NER mutants. Again we found that *rad-23; csb-1* double mutants were more sensitive than either *rad-23* or *csb-1* single mutant alone and were comparable to general NER mutants (Figure 5C). Although *rad-23* mutant L1 larvae did not show increased lethality, they did appear to develop slightly slower in response to UV irradiation (data not shown). Next, we also tested the recently available *xpc-1* mutant. *xpc-1* single and *rad-23; xpc-1* double mutants did not show enhanced UV sensitivity compared to wild type animals (Figure 5D). *rad-23; xpc-1* double mutants even showed a mild but reproducible increase in UV survival. Other functions of *rad-23*, besides NER [32], [33], might account for this observation, although at the moment we do not understand how these might stimulate UV survival. Importantly, *xpc-1; csb-1* double mutants showed extreme UV sensitivity comparable to that of general NER mutants and the *rad-23; csb-1* double mutant (Figure 5D). Similar results were obtained using older larval stages and young adults instead of L1 larvae (data not shown). Together, these results (summarized in Table 1) suggest that in contrast to germ cells, TCR is the major NER pathway acting in juvenile and adult *C. elegans* tissues to counteract the effects of UV irradiation (Table 1, Figure 5E). The GGR pathway seems to act partially redundantly to the TCR pathway.

The observed difference in UV survival of *rad-23/xpc-1* and *csb-1* during development suggests that as a germ cell grows to become an L1 larva, a switch occurs that favors the dependence on one pathway over the other. To test at which developmental stage *csb-1/TCR* becomes the primary UV survival pathway instead of *rad-23/GGR*, we collected eggs from adult animals by hypochlorite treatment and irradiated these at different time points after collection (Figure S1C). We found that in early eggs, *rad-23* function is still essential for optimal UV survival (Figure 5F; 1 hr, 20 J/m^2^), similar to germ cells and embryos. However, in time *rad-23* function became gradually dispensable while *csb-1* function was more and more essential for optimal UV survival (Figure 5G; 4 and 8 hr, 40 J/m^2^). Note that during later time points slightly higher UV doses had to be used due to the fact that early embryos are more UV sensitive than later stage embryos. This phenomenon might be due to growth causing less UV penetrance or higher tolerance of transcription and replication blocking lesions [30]. Irradiation of eggs collected by egg laying gave similar results as eggs collected by hypochlorite treatment (data not shown; [30]). In summary, these results suggest that during embryogenesis, before hatching, GGR gradually becomes less and TCR becomes more important for *C. elegans* to cope with the toxic effects of UV exposure.

### Novel chromatin remodelers in UV damage response

The developmental difference between TCR- and GGR-dependent UV-sensitivity of *C. elegans* suggests that developmental-stage dependent regulatory genes specifically involved in either pathway could be identified using *C. elegans*. Recently, we have successfully used *C. elegans* to show that Heterochromatin Protein 1 (HP1), represented by *hpl-1* and *hpl-2* in *C. elegans*, is involved in the UV-DDR [34], suggesting a role for chromatin condensation status in UV survival. This implies that proteins involved in chromatin dynamics, e.g. chromatin remodeling and epigenetics, may be implicated in the UV-DDR. These proteins are expected to play important roles in controlling the efficiency of DNA repair, by regulating the access to DNA as well as checkpoint signaling associated with DNA repair [35]. CSB itself is an ATP-dependent chromatin remodeling factor, which is thought to alter nucleosome structure to enable repair [36], [37]. In yeast, fruit flies and mammals, several different ATP-dependent chromatin remodeling complexes, e.g. the SWI/SNF, the ISWI, the NuRD, the CHD and the INO80 families, have been identified, some of which have been implicated in the DDR [35], [36]. To test whether these remodeling complexes are involved in the developmental stage-dependent UV-DDR in *C. elegans*, we set up a screen in which we systematically tested L1 larvae UV sensitivity of animals in which subunits of these major remodeling complexes or genes carrying motifs predicted to be involved in ATP-dependent chromatin remodeling were knocked down either by mutation or RNAi (Table S1).

**Figure 6:**
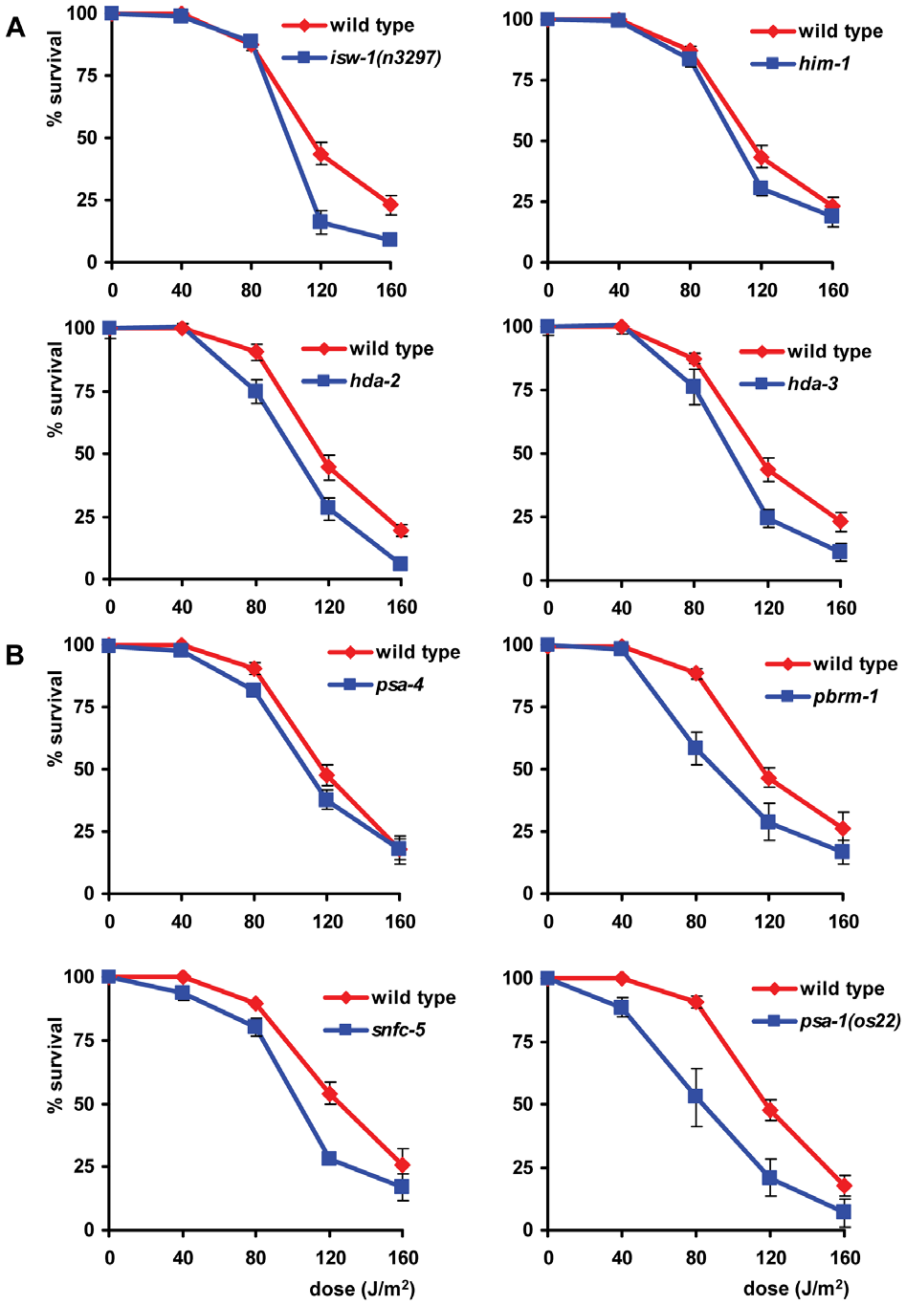
ISWI and SWI/SNF chromatin remodeling function in L1 larvae UV sensitivity. (A) The L1 larvae UV survival of animals in which members of the ISWI family of chromatin remodelers are knocked down by mutation. (B) The L1 larvae UV survival of animals in which members of the SWI/SNF family of chromatin remodelers are knocked down by mutation. Each line represents the mean of at least two independent experiments, each performed in duplicate (typically, n>40). Data for *isw-1*, *him-1*, *hda-2*, *hda-3*, *pbrm-1*, *snfc-5* and *psa-1* were normalized because without UV irradiation these mutants already show minor larval arrest. Error bars denote the s.e.m.

UV survival of L1 larvae in which proteins of the NuRD, the CHD and the INO80 chromatin remodeling family were knocked down closely mimicked that of wild type larvae (data not shown), suggesting no involvement in the UV-DDR. In contrast, knockdown of four proteins of the ISWI family and four proteins of the SWI/SNF family resulted in increased UV-sensitivity (Table 1, Figure 6). We tested two partial loss-of-function alleles of the ISWI/SMARCA5 chromatin remodeling ATPase subunit *isw-1* [38]. *isw-1*(n3297) animals showed reproducible sensitivity to UV irradiation (Figure 6A), but *isw-1(n3294)* animals did not (Figure S2A). Surprisingly, *isw-1*(n3297) carries a missense mutation within a non-conserved region of the gene while *isw-1(n3294)* encodes a missense mutation in a conserved DEXD/H box helicase domain required for chromatin remodeling activity [38]. Since *isw-1* null mutants are not viable, we additionally knocked down *isw-1* using RNAi and confirmed that *isw-1* functions in the UV-DDR (Figure S2B). Furthermore, deletion alleles of *hda-2(ok1479)* and *hda-3(ok1991)*, which represent orthologs of human class I histone deacetylase [39], and mutation of *him-1*, the *C. elegans* ortholog of human cohesin protein SMC1, which are all found in complex with human ISWI/SMARCA5 [40], also increased UV-sensitivity (Figure 6A). To confirm the significance of these findings, we reproduced the observed UV sensitivities in multiple independent experiments. Knockdown of *hda-2* and *hda-3* by RNAi was also attempted, but was found to produce variable results (data not shown), possibly because efficacy of the RNAi was not always optimal. As the e879 allele used for *him-1* was described to be temperature-sensitive [41], we additionally tested *him-1* mutants at 25°C and found them to be more UV sensitive than at 20°C (Figure S3A). This increased UV sensitivity at the restricted temperature further confirmed that this gene is indeed implicated in the UV-DDR.

Next, we tested animals carrying a temperature sensitive missense mutation (*os13*) in the SWI2/SNF2 chromatin remodeling ATPase subunit *psa-4* [42], a putative ortholog of human BRM/SMARCA2. Animals tested at a permissive temperature (20°C) were found to be mildly sensitive to UV (Figure 6B), whereas animals tested at a nonpermissive temperature (25°C) showed a strongly enhanced UV sensitivity (Figure S3B). Additionally, mutations in other subunits of SWI/SNF remodeling complexes, e.g. the SMARCC1 ortholog *psa-1(os22* and *ku355*; [42], [43]), the PBRM1 ortholog *pbrm-1(tm415)* and the SMARCB1 ortholog *snfc-5(ok622)* also increased UV-sensitivity (Figure 6B, Figure S2C). As both *psa-1* alleles were described to be temperature-sensitive, we tested both alleles at 25°C and found them to confer even stronger UV-hypersensitivity than at 20°C (Figure S3B). The UV hypersensitivities of all SWI/SNF mutants were reproduced in multiple, independent experiments, corroborating their significance. Furthermore, knockdown of *pbrm-1* and *snfc-5* using RNAi also mildly increased UV sensitivity (data not shown). In summary, these results implicate the ISWI and SWI/SNF chromatin remodeling complexes in the UV-DDR of *C. elegans*. Mutation or RNAi-mediated knockdown of other members of both ATP-dependent chromatin remodeling complexes (Table S1) had no effect, possibly because RNAi was not efficient or because these factors do not play a role in the UV-DDR. Involvement of some factors could not be tested due to lethality.

**Figure 7:**
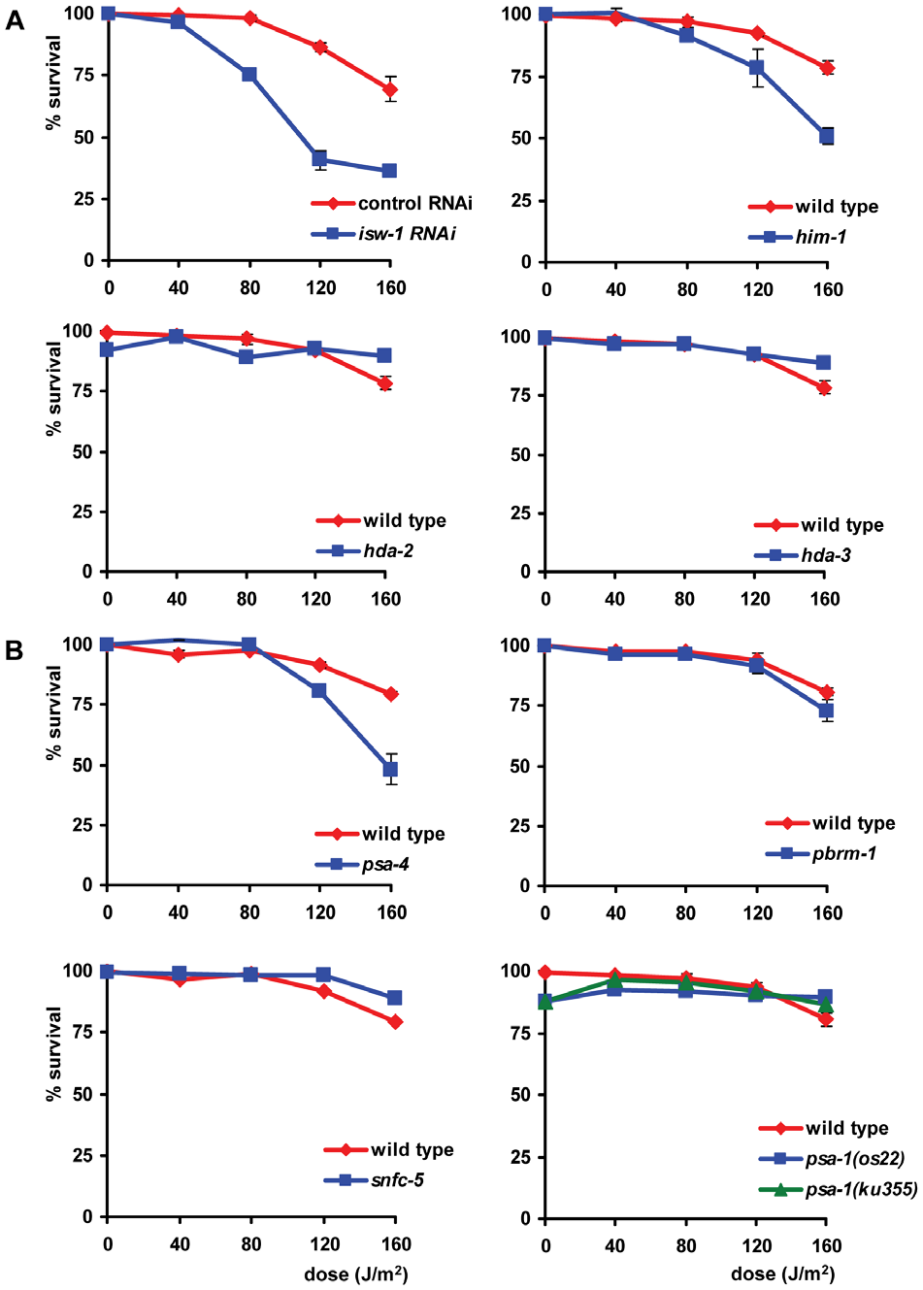
Involvement of ISWI and SWI/SNF chromatin remodeling in germ cell and embryo UV sensitivity. (A) The germ cell and embryo UV survival of animals in which members of the ISWI family of chromatin remodelers are knocked down by mutation or RNAi. (B) The germ cell and embryo UV survival of animals in which members of the SWI/SNF family of chromatin remodelers are knocked down by mutation. Each line represents the mean of at least two independent experiments (typically, n>40 eggs). Data for *him-1*, *psa-4* and *pbrm-1* were normalized because without UV irradiation these mutants already produce unviable eggs. Error bars denote the s.e.m.

In addition to the L1 larvae survival experiment, we tested whether the eight identified genes are also involved in the UV-DDR of germ cells and embryos. Since both *isw-1* mutants did not lay sufficient eggs on a regular basis, we tested *isw-1* involvement using RNAi. Knockdown of the *isw-1* and *psa-4* ATPase subunits of ISWI and SWI/SNF chromatin remodeling complexes, and of the cohesin member *him-1*, rendered germ cells sensitive to UV (Table 1, Figure 7). However, mutation of the other ISWI and SWI/SNF subunits had no significant effect on UV survival. These results suggest that ISWI and SWI/SNF chromatin remodeling activity is involved in UV survival of germ cells and embryos, but the response in germ cells seems to involve other subunits than the response in L1 larvae.

### Genetic interactions among *isw-1*, *pbrm-1*, *rad-23*, and *csb-1*

**Figure 8:**
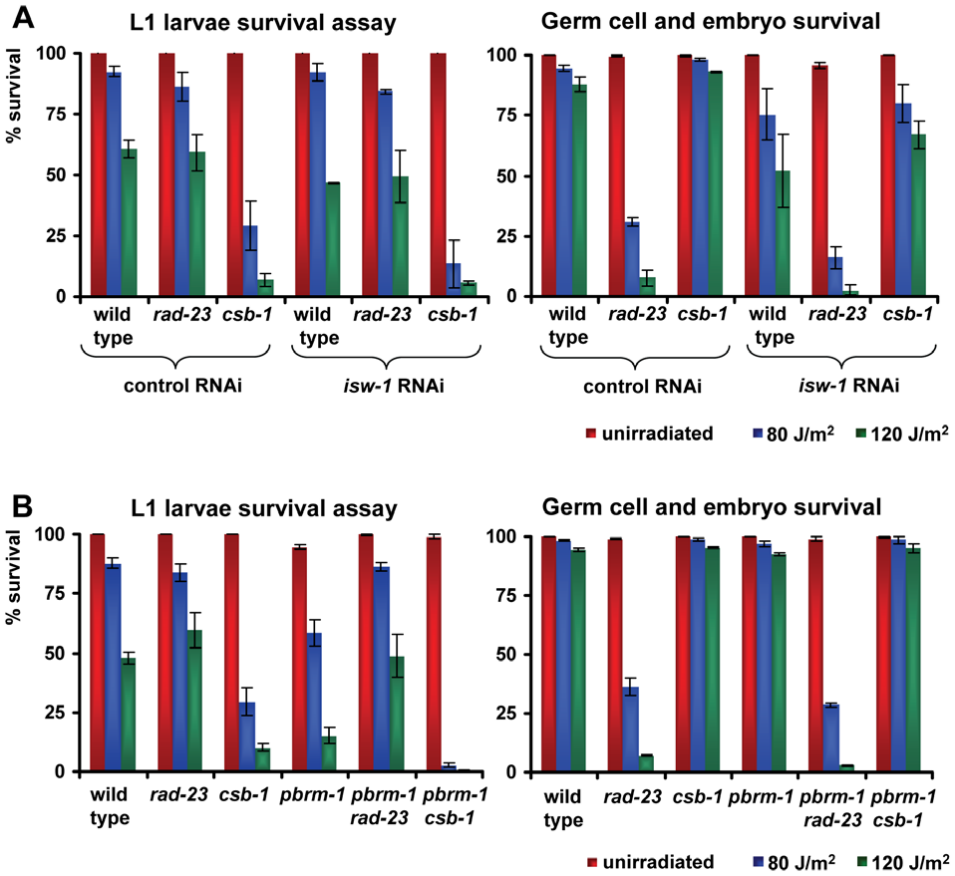
Genetic interactions of *isw-1* and *pbrm-1* with *rad-23* and *csb-1*. (A) L1 larvae and germ cell and embryo UV survival of wild type, *rad-23* and *csb-1* animals grown on control or *isw-1* RNAi food. (B) L1 larvae and germ cell and embryo UV survival of wild type, *rad-23*, *csb-1*, *pbrm-1*, *pbrm-1; rad-23* and *pbrm-1; csb-1* animals. Each line represents the mean of at least two independent experiments (typically, n>40). Error bars denote the s.e.m.

The specific UV sensitivity of L1 larvae but not germ cells caused by knockdown of certain chromatin remodeling genes suggests these genes might be involved in TCR but not GGR. If this is the case, knockdown of these genes in a GGR-deficient background could lead to an even more pronounced UV sensitivity, similar as observed for the *rad-23; csb-1* double mutants. Likewise, genes that affect UV sensitivity in L1 larvae as well as germ cells might be generally involved in NER, in both TCR and GGR. Inactivation of these genes in a GGR- or TCR-deficient background should not lead to increased UV sensitivity. To test this, we inactivated *isw-1*, which affects sensitivity in germ cells and L1 larvae, and *pbrm-1*, which only affects L1 larvae sensitivity, in *rad-23* and *csb-1* mutants. RNAi-mediated knockdown of *isw-1* in *rad-23* and *csb-1* animals did not lead to significantly enhanced UV sensitivity compared to the respective controls, in both the L1 as well as the germ cell and embryo survival assay (Figure 8A). Only a mild, but reproducible increase in UV sensitivity was observed in the germ cell and embryo sensitivity of *rad-23* mutants in which *isw-1* was knocked down. Most of these results, however, are in line with the idea that *isw-1* has a general regulatory role in the UV-DDR but not specifically in either TCR or GGR.

Next, we created double mutants for *pbrm-1* and *rad-23* or *csb-1* and compared their UV sensitivity to respective controls (Figure 8B). This showed L1 larvae UV sensitivity of *pbrm-1; rad-23* double mutants was comparable to *rad-23* single mutants and less severe to that of *pbrm-1* single mutants. Unexpectedly, L1 larvae UV sensitivity of *pbrm-1; csb-1* double mutants was enhanced compared to *csb-1* and *pbrm-1* single mutants. These results, which were reproduced in independent experiments, suggest in L1 larvae *rad-23* is epistatic to *pbrm-1*, while *pbrm-1* and *csb-1* act synergistically to protect against UV exposure. In germ cells and embryos no difference in UV sensitivity between *pbrm-1; rad-23* and *pbrm-1; csb-1* double mutants and their respective controls was observed. In conclusion, although our results clearly indicate a function for *pbrm-1*, *isw-1* and the other chromatin remodeling genes in the UV-DDR, their precise mode of action is still ambiguous and might not be simply confined to either TCR or GGR.

## Discussion

### Nucleotide excision repair in *C. elegans*

**Figure 9:**
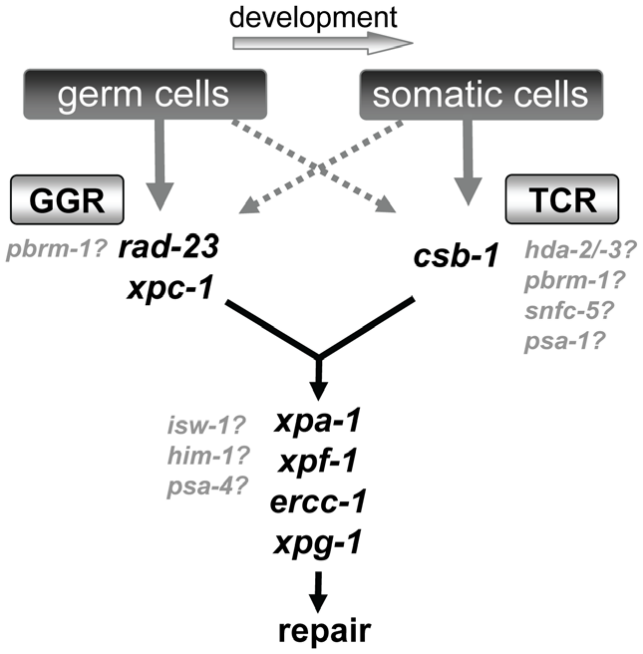
Model of NER function in germ cells and somatic cells. Our results suggest in germ cells and embryos GGR is the major pathway contributing to repair and survival of UV induced DNA damage, while TCR has a secondary role. As embryos develop to become L1 larvae, this role of GGR becomes less important. From L1 larvae to adults, TCR is the major pathway involved in survival of UV irradiation, whereas GGR is still active but has a secondary role. The precise function of the chromatin remodeling genes that are implicated in the UV-DDR is not yet clear. Some may function to modulate TCR, but others may have a more general regulatory function.

The genetic analysis presented in this paper strongly suggests that NER functions mechanistically similarly in the nematode *C. elegans* as it does in mammals. We and others [17]–[20], [22] find that functional loss of core NER factors renders animals hypersensitive to UV light. Similar as in mammals, NER can be initiated by two distinct pathways, GGR and TCR, which depend on *rad-23/xpc-1* and *csb-1*, respectively. The clear difference between *rad-23/xpc-1* and *csb-1* UV sensitivities during development and the enhanced UV sensitivity in *rad-23/xpc-1; csb-1* double mutants makes it unlikely that the RAD-23 and XPC-1 proteins are involved in both TCR and GGR. Therefore, *C. elegans* NER seems distinct from NER in budding yeast, where RAD23 and RAD4 (yeast orthologs of hHR23 and XPC, respectively) play a role in TCR as well [44], [45]. Importantly, we observe that the involvement of GGR and TCR in *C. elegans* is developmentally regulated and differs between germ and somatic cells (Table 1; Figure 9). This developmental regulation was not noticed before in eukaryotes, but might be important for understanding the etiology of different mammalian syndromes associated with NER deficiencies. Following our analysis of the UV-DDR in *C. elegans*, we identify eight genes involved in ATP-dependent chromatin remodeling that function in the UV-DDR, depending on the developmental stage. Together, our data suggests *C. elegans* is a powerful model organism to study UV-induced DNA repair and to identify novel genes involved in this process.

### Global Genome and Transcription Coupled Repair in *C. elegans*

We provide evidence that in germ cells, oocytes and early embryo's GGR is the main DNA repair pathway conveying UV resistance. Our analyses of UV-survival, CPD repair, pachytene cell apoptosis and pachytene stage exit all indicate that *rad-23* and *xpc-1* are necessary and sufficient for germ cells to overcome the effects of UV irradiation. However, it is not exactly clear how UV irradiation of germ cells leads to the embryonic death that is measured in the germ cell and embryo survival assay (Figure 2 and S1A). It is tempting to speculate that the lack of UV-induced apoptosis and defective pachytene stage exit leads to embryonic death. However, animals lacking the *C. elegans* p53 ortholog also show no UV-induced apoptosis, but have wild type levels of embryonic UV survival [22]. Furthermore, animals carrying a gain-of-function mutation (*n1950*) in the core cell death pathway gene *ced-9*, also do not show radiation-induced apoptosis [27] and do not show enhanced UV-induced embryonic lethality (unpublished results). These observations indicate that lack of apoptosis and embryonic death are not necessarily linked.

Our results confirm previous observations that in pachytene cells lacking functional XPA-1 apoptosis is not induced after irradiation [22]. This might imply that active NER is necessary to signal the presence of DNA damage to the apoptotic machinery, via the generation of NER-intermediates such as single stranded DNA [46]. Analysis of the *rad-23* and *csb-1* single and double mutants suggests that GGR or TCR alone is sufficient to induce apoptosis, although via TCR, e.g. in the *rad-23* mutant, it seems to be slightly less efficient. Lack of functional GGR and TCR together inhibits induction of apoptosis. These results contrast the apoptotic response observed in cultured mammalian cells, which undergo increased apoptosis after irradiation when NER, and specifically TCR in differentiated cells, is impaired [47]–[49]. In these cells it is believed that persistence of damage in the transcribed strand of active genes triggers apoptosis. In undifferentiated mouse embryonic stem cells UV-irradiation induces apoptosis in NER-deficient XP-A cells but not in GGR-deficient XP-C cells [47]. Thus, it might be that in undifferentiated cells, similar to *C. elegans* germ cells, a trigger derived from GG-NER or a repair intermediate is necessary to set off an apoptotic response, contrary to the mainly TCR-driven apoptotic response of differentiated cells. An alternative explanation for the lack of apoptosis in NER deficient *C. elegans* germ cells might be that UV causes defects in cell cycle progression. Because of this, cells might not reach the late pachytene stage in which they can become apoptotic.

Our results indicate that in *C. elegans* the involvement of GGR and TCR in survival of UV-induced DNA damage changes during development (Figure 9). A similar developmental change was described for the homologous recombination (HR) and non-homologous end-joining (NHEJ) repair pathways in *C. elegans* [50]. The error-free HR pathway is mainly active in germ cells and dividing somatic cells, while the error-prone NHEJ pathway becomes predominantly active in non-dividing somatic cells. This difference is probably to ensure that the genome integrity of germ cells and dividing cells is maintained, while genomic damage in non-dividing cells can be tolerated. Similarly, GGR may act in germ cells to ensure that the entire genome is free of lesions. TCR is only necessary to maintain active genes in non-dividing somatic cells. These findings exemplify the advantage of using *C. elegans* as *in vivo* tool to study the DNA repair response and are in line with similar observations in mammalian cells. Terminally differentiated human neurons appear to lose the ability to repair DNA lesions throughout the genome whereas they retain the ability to repair active genes [51]. Furthermore, in undifferentiated mouse embryonic stem cells the contribution of GGR to UV survival is larger than that of TCR, whereas in partially differentiated mouse embryonic fibroblasts the contribution of TCR is larger than that of GGR [47]. Although GGR is the major pathway contributing to survival in germ cells, we observed that TCR is also active but not essential for survival in these cells. Vice versa, in later developmental stages TCR is essential for survival, while GGR is also active but not essential for survival. The differences in activity of both repair pathways in later stages correlates to previous observations showing that in adult *C. elegans* highly transcribed and poorly transcribed genes are both repaired, although highly transcribed genes more efficient [52].

It is still unclear what causes the developmental switch from GGR to TCR. A possible mechanism might be that the switch occurs simultaneously with the onset of transcription in embryos, since TCR depends on transcription. However, transcription takes place in pachytene germ cells as well [29] and is initiated in the embryo already several hours before the *csb-1* UV sensitivity becomes visible. A second mechanism might be that the switch is linked to proliferation, as the *csb-1* UV sensitivity becomes visible when most cell divisions in the embryo have been completed [53]. However, oocytes, which depend on *rad-23*, do not divide, while L1 larvae, in which cell division resumes, depend on *csb-1*. A third mechanism might be the availability of RAD-23 and CSB-1 at the site of damage. Although both *rad-23* and *csb-1* appear to be expressed in all cells throughout development (data not shown; [17], [52]), there might be a delicate balance between RAD-23 and CSB-1 availability at the site of damage which is for instance influenced by chromatin-dependent accessibility of DNA. This hypothesis, however, does not correlate with the fact that the UV-DDR depends on *rad-23* in all different cells of the germ line, while these cells differ significantly with regard to chromatin compaction. Finally, it might simply be that different processes are involved in survival and cell death when comparing germ cells to later stage somatic cells. Part of the UV sensitivity may result from direct interference of photolesions with vital processes such as transcription and replication. However, UV sensitivity may also be partially caused by extensive chromosomal aberrations which are caused by UV irradiation in *C. elegans* [54]. Germ cells might die from UV irradiation because global genome DNA damage, which is not repaired in a *rad-23* genetic background, interferes with meiotic progression and early cell divisions. Later stage somatic cell types probably arrest due to block of transcription, which is persistent in a *csb-1* genetic background [20].

### ISWI and SWI/SNF chromatin remodeling in the UV damage response

Recent studies have highlighted the role of (ATP-dependent) chromatin remodeling in DNA repair, mainly focusing on the double-strand break response [35], [36]. Using a dedicated genetic screen we identified eight genes implicated in chromatin remodeling whose involvement in the UV-DDR was unknown or at least ambiguous. Several lines of evidence suggest these genes genuinely function in the UV-DDR, instead of indirectly influencing UV survival because of their involvement in other processes such as transcription. First, inactivation of five genes caused UV hypersensitivity specifically in L1 larvae while inactivation of three other genes also caused germ cell hypersensitivity (Table 1). This specific difference between L1 and germ cell UV response would not be expected if UV hypersensitivity resulted indirectly from the impairment of other processes. Second, many other genes whose knockdown probably causes pleiotropic phenotypes (see Table S1) were not found to be involved in the UV response. This also argues for a specific role of the eight identified chromatin remodeling genes in the UV-DDR. Finally, comparisons to literature and other DNA repair mechanisms suggest these genes might facilitate access of proteins to DNA or be involved in DNA damage signaling (see discussion below). The mild UV hypersensitivity of the chromatin remodeling mutants, which contrasts the severe hypersensitivity of NER mutants, is in line with such a regulatory role.

We identified four genes implicated in ISWI-dependent chromatin remodeling, *isw-1*, *hda-2*, *hda-3* and *him-1*. Mutation of *him-1* was shown before to cause UV sensitivity [21], while *isw-1*, *hda-2* and *hda-3* were also identified in previous damage response screens [55], [56]. The human *isw-1* ortholog SMARCA5 is part of a chromatin remodeling complex that includes the *hda-2/-3* ortholog HDAC1 and the cohesin subunit *him-1* ortholog SMC1 [40]. Therefore, our findings suggest that an ISWI/cohesin complex involving these proteins is involved in the UV-DDR. However, since these proteins participate in several different other protein complexes, they might regulate the UV response independently of each other. This is suggested by the fact that *isw-1* and *him-1* loss-of-function causes sensitivity in germ cells, embryo's and L1 larvae, whereas *hda-2* and *hda-3* loss-of-function only affects L1 larvae. Alternatively, it could be that different ISWI/Cohesin complexes regulate different aspects of the UV-damage response that differ between germ cells and somatic cells and only involve *hda-2*/*hda-3* in somatic cells (Figure 9). Several previous observations support a role for ISWI/Cohesin in the UV-DDR. For instance, the *Drosophila* ACF complex, containing the *isw-1* ortholog ISWI, was found to facilitate NER in dinucleosomal DNA *in vitro* [57]. Furthermore, SMC is known to be phosphorylated following ionizing or UV irradiation and is thought to play a role in the S-phase checkpoint response in mammalian cells [58], [59]. The evolutionary conserved function of ISWI/Cohesin activity in different DNA damage responses in different species suggests it is involved in one or more steps which are common among DNA damage pathways and possibly involve slightly different complexes: (i) ISWI and/or cohesin may function to mediate a DNA damage induced checkpoint response and (ii) ISWI and/or cohesin may function to facilitate efficient repair by altering chromatin structure. Follow-up functional studies will be required to explore the exact molecular role of ISWI/cohesin in the UV-DDR.

Our analysis further implicated four genes involved in SWI/SNF mediated chromatin remodeling in the UV-DDR. *pbrm-1*, *psa-1* and *snfc-5*, orthologs of human PBRM1, SMARCC1 and SMARCB1, respectively, only showed UV sensitivity when irradiated as L1 larvae, similar to *hda-2* and *hda-3*. Since the L1 larvae survival assay seems specific for TCR, this would suggest that these genes are specifically involved in TCR or a TCR-associated process (Figure 9). However, our genetic analysis of *pbrm-1; rad-23* and *pbrm-1; csb-1* double mutants suggests that *pbrm-1* acts in parallel to *csb-1* but not *rad-23* in L1 larvae. To clarify these seemingly contradicting results, more detailed follow-up experiments to determine the precise function of *pbrm-1* are necessary. *psa-4*, ortholog of human BRM/SMARCA2, showed also UV sensitivity in the germ line, indicating that it might have a more general role in the UV-DDR. Possibly, different ATP-dependent chromatin remodeling complexes play a role during TCR compared to GGR, or throughout development, while they may share some of the same subunits. In mammals, several different SWI/SNF-like complexes have been identified containing either BRM/SMARCA2, the ortholog of *psa-4* [42], or BRG1 as ATPase subunit. Furthermore, involvement of other subunits such as SMARCC1 (*psa-1*), PBRM1 (*pbrm-1*) and SMARCB1 (*snfc-5*) also differs between different SWI/SNF complexes. SWI/SNF chromatin remodeling has been implicated in the UV-DDR before, but the exact mechanism by which it functions remain unknown. Mammalian cells lacking SMARCB1 or the BRM-paralog BRG1 are hypersensitive to UV irradiation, possibly because SWI/SNF functions in the checkpoint response [60], [61]. Yeast SWI/SNF chromatin remodeling, on the other hand, was shown to stimulate excision repair *in vitro* and in cells, possibly because of rearrangement of chromatin at a damaged site to allow repair [62], [63]. Therefore, it remains unclear whether SWI/SNF chromatin remodeling directly participates in the repair of a lesion or whether it modulates the checkpoint response, or whether it functions in both processes but involves complexes of different composition. We expect that the identification of specific SWI/SNF genes involved in the UV-DDR will lead to a better understanding of the role of SWI/SNF in the DNA repair mechanism.

In summary, our analysis showed that *C. elegans* is especially well suited to genetically dissect genes and pathways involved in the UV-DDR at different stages of development. Based on the observed evolutionary conserved role of UV-DDR in *C. elegans*, it is expected that further analysis using the nematode will increase our understanding of how this response is organized in living organisms.

## Materials and Methods

### *C. elegans* alleles, RNAi

All strains were cultured according to standard methods [64]. Alleles used were *csb-1(ok2335)*, *ercc-1(tm2073)*, *hda-2(ok1479)*, *hda-3(ok1991)*, *him-1(e879)*, *him-9(e1487)*, *isw-1(n3294)*, *isw-1(n3297)*, *pbrm-1(tm415)*, *psa-1(ku355)*, *psa-1(os22)*, *psa-4(os13)*, *rad-23(tm2595)*, *snfc-5(ok622)*, *xpa-1(ok698)*, *xpc-1(tm3886)* and *xpg-1(tm1670)*. *snfc-5*, *xpa-1*, *xpc-1*, *ercc-1*, *rad-23* and *csb-1* mutants were backcrossed four times, *pbrm-1* was backcrossed three times. Double mutants were genotyped using PCR (primer sequences available upon request). RNAi bacteria were obtained from the *Caenorhabditis elegans* RNAi feeding library (Geneservice). Control RNAi was vector pPD129.36 (a gift from A. Fire).

### Germ cell and embryo survival assay

To measure UV sensitivity of germ cells and early embryos, staged young adults were washed and transferred to empty agar plates (Figure S1A). Next, animals were irradiated at the indicated dose using two Philips TL-12 (40W) tubes emitting UVB light, after which they were transferred to plates plated with OP50 bacteria. Following a 24 hr recovery period, animals were allowed to lay eggs for 2–3 hrs on fresh 6 cm plates containing food. In each experiment, for each dose 6 plates containing 3–5 adults per plate were used. The number of eggs laid was determined and 24 hours later the number of unhatched eggs, to calculate the survival percentage.

### Egg survival and L1 larvae survival assay

To measure UV sensitivity of eggs or L1 larvae, eggs were collected from gravid adult animals by hypochlorite treatment and transferred to fresh plates seeded with HT115(DE3) bacteria (Figure S1B and S1C). HT115(DE3) bacteria were specifically used because these bacteria form a uniform thin lawn on NGM plates, which increases reproducibility of the survival assay, as the thicker lawn formed by OP50 bacteria was found to partially shield *C. elegans* from UV irradiation. We did not observe any typical effects using HT115(DE3) bacteria related to the UV sensitivity of animals. To measure egg survival (Figure 5D), animals were irradiated at indicated time points following hypochlorite treatment. The number of unhatched and hatched eggs was determined 24 hours later to calculate the survival percentage. To measure L1 larvae survival, animals were irradiated 16 hrs (at 20°C) after hypochlorite treatment. Animals that developed beyond the L2 stage (survivors) and animals that arrested development at the L1/L2 stage were counted to determine survival percentage. For experiments performed at 25°C (Figure S3), animals were cultured at 20°C and transferred to 25°C 45 minutes before irradiation. Hypochlorite treatment had no effect on survival rates (data not shown) and similar results were obtained by regular egg laying. Statistical analysis was performed using a one-way ANOVA test.

### Immunofluorescence and DAPI staining

To visualize CPD DNA damage, gonads were extruded by cutting the heads and tails of young adult animals using a fine gauge needle. Gonads were fixed in 3% paraformaldehyde, 0.1% Triton X-100 for 15 minutes, washed and permeabilized 2 times 10 minutes in PBS, 0.1% Triton X-100. Next, gonads were incubated for 5 minutes in PBS, 0.07 M NaOH, to denature DNA. Gonads were then washed in PBS, 0.5% BSA, 0.15% glycin and incubated >2 hrs with CPD antibody (Cosmo Bio Co.) in PBS, 0.5% BSA, 0.15% glycin. Subsequently, animals were washed 2 times 10 minutes in PBS, 0.1% Triton X-100 and incubated >2 hrs with Alexa488 fluorescent secondary antibody (Molecular Probes). Finally, animals were mounted on a glass slide using Vectashield with DAPI (Vector laboratories). For DAPI staining, animals were fixed, permeabilized and mounted on a slide using Vectashield with DAPI.

### Microscopy and germ line apoptosis

Images in Figure 3C and Figure 4 were acquired using a Zeiss LSM 510 META confocal microscope. Images in Figure 3B 1 were acquired using a Zeiss Axio Imager.Z1 and Nomarski optics. To determine germ line apoptosis, staged young adult animals were irradiated using 160 J/m^2^ UVB. Six hours later germ cell apoptosis was scored using Nomarski optics.

## Supporting Information

Figure S1 Schematic representations of UV survival assays. (A) For the germ cell and embryo survival assay, adult animals were irradiated on empty plates and transferred to food containing plates. Following 24 hrs of recovery, animals were allowed to lay eggs for several hours. Survival was scored by counting surviving and dead eggs. (B) For the L1 larvae survival assay, eggs were transferred to plates containing a thin layer of HT115(DE3) bacteria. Following hatching, L1 larvae were irradiated and survival scored by counting surviving animals and arrested animals. (C) For the egg survival assay, eggs were transferred to plates containing a thin layer of HT115(DE3) bacteria and irradiated at different time points. Survival was scored by counting surviving and dead eggs.
(0.29 MB TIF)

Figure S2 *isw-1* and *psa-1* knockdown induce L1 larvae UV sensitivity. (A) The *isw-1(n3294)* allele does not confer increased sensitivity to UV irradiation, but (B) RNAi induced knockdown of *isw-1* does increase UV sensitivity. (C) In addition to the *os22* allele, the *ku355* allele of *psa-1* confers increased UV sensitivity. Each line represents the mean of at least two independent experiments, each performed in duplicate (typically, n>40). Data for *psa-1* was normalized as animals show minor larval arrest without UV irradiation. Error bars denote the s.e.m.
(0.14 MB TIF)

Figure S3 L1 larvae UV sensitivity at 25°C of *him-1*, *psa-4* and *psa-1* mutants. UV sensitivity was tested at 25°C. Shown is the L1 larvae UV survival of animals carrying temperature-sensitive mutations in *him-1(e879)* (A), *psa-4(os13)* and *psa-1*(*os22* and *ku355*) (B). Each line represents the mean of at least two independent experiments, each performed in duplicate (typically, n>40). Data for *psa-4* and *psa-1* were normalized because without UV irradiation these mutants already show some larval arrest. Error bars denote the s.e.m.
(0.14 MB TIF)

Table S1 Genes tested for effect on L1 survival after UV irradiation. Genes tested for their involvement in L1 larvae survival after UV irradiation. If available, mutant alleles were tested. If alleles resulted in lethality or were not available, we applied RNAi to knockdown gene function. Protein domain SNF2 stands for SNF2 family N-terminal domain (Pfam domain PF00176), ARID stands for ARID/BRIGHT DNA binding domain (Pfam domain PF01388).
(0.08 MB DOC)
