# Supplementary figures and images for: Involvement of Global Genome Repair, Transcription Coupled Repair, and Chromatin Remodeling in UV DNA Damage Response Changes during Development
Source: PLoS Genet. 2010 May 6;6(5):e1000941. doi: 10.1371/journal.pgen.1000941 (PMC2865526; doi:10.1371/journal.pgen.1000941)

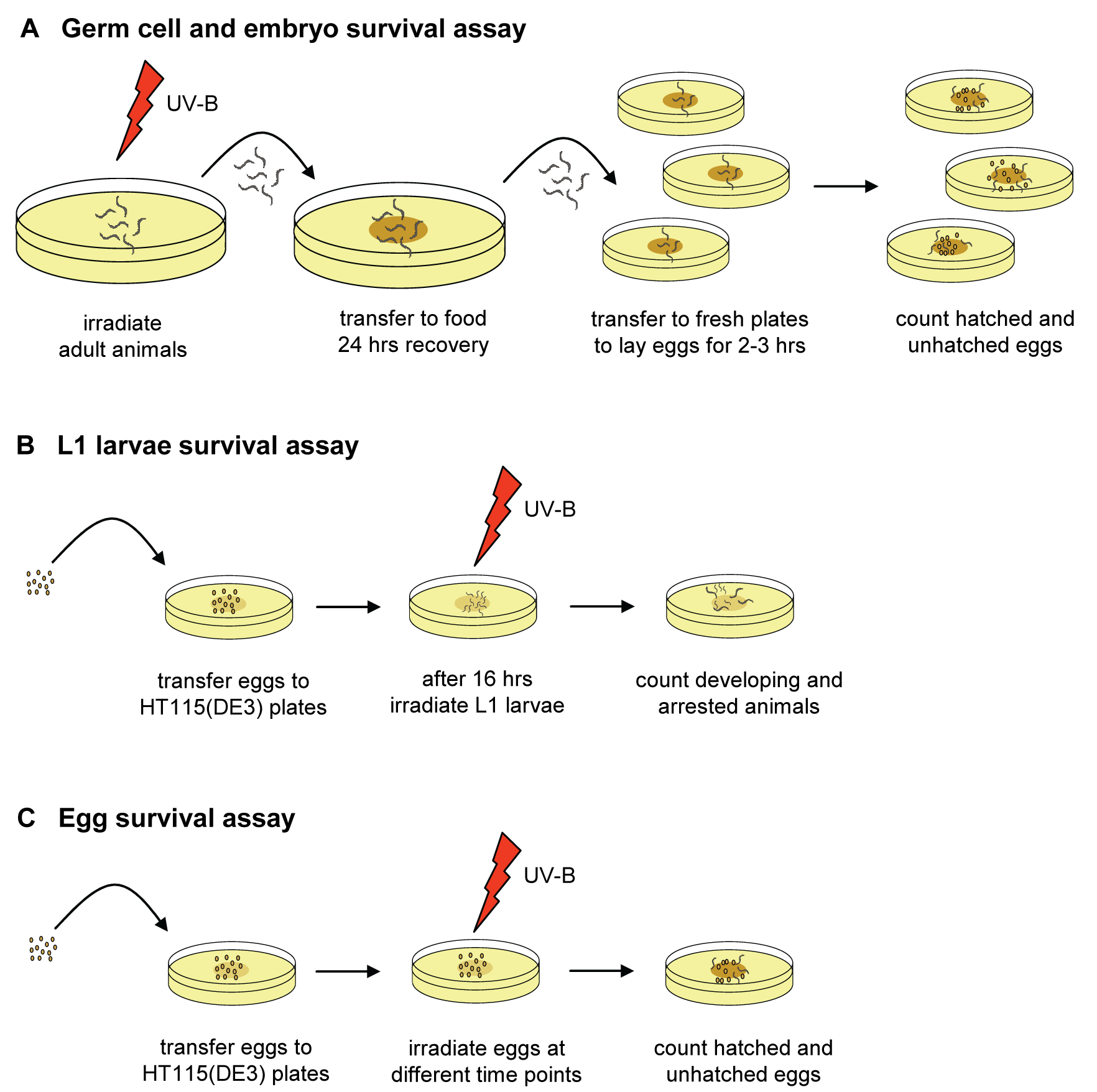

Supplement: Figure S1 — Schematic representations of UV survival assays. (A) For the germ cell and embryo survival assay, adult animals were irradiated on empty plates and transferred to food containing plates. Following 24 hrs of recovery, animals were allowed to lay eggs for several hours. Survival was scored by counting surviving and dead eggs. (B) For the L1 larvae survival assay, eggs were transferred to plates containing a thin layer of HT115(DE3) bacteria. Following hatching, L1 larvae were irradiated and survival scored by counting surviving animals and arrested animals. (C) For the egg survival assay, eggs were transferred to plates containing a thin layer of HT115(DE3) bacteria and irradiated at different time points. Survival was scored by counting surviving and dead eggs. (0.29 MB TIF) [file pgen.1000941.s001.tif]

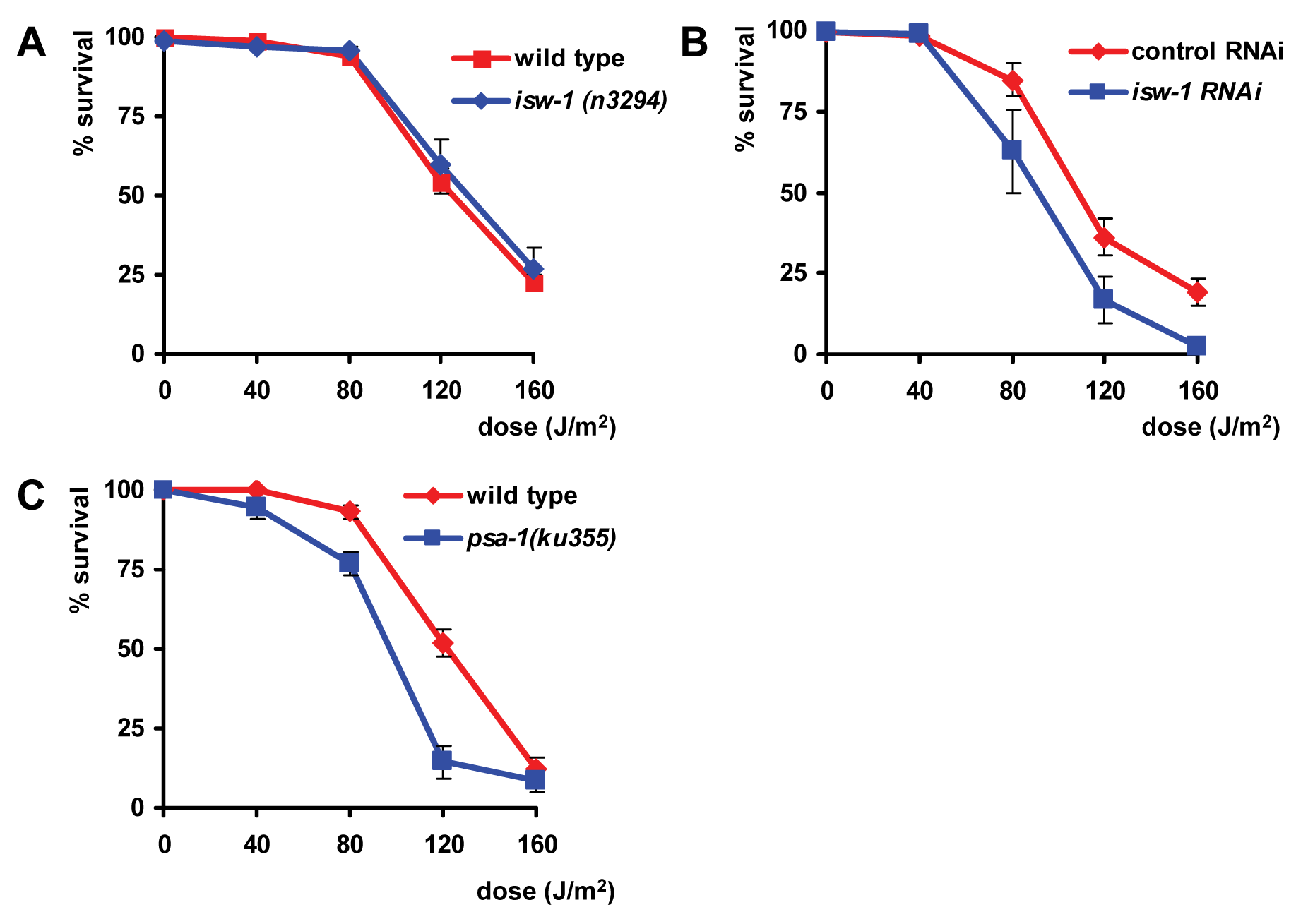

Supplement: Figure S2 — isw-1 and psa-1 knockdown induce L1 larvae UV sensitivity. (A) The isw-1(n3294) allele does not confer increased sensitivity to UV irradiation, but (B) RNAi induced knockdown of isw-1 does increase UV sensitivity. (C) In addition to the os22 allele, the ku355 allele of psa-1 confers increased UV sensitivity. Each line represents the mean of at least two independent experiments, each performed in duplicate (typically, n>40). Data for psa-1 was normalized as animals show minor larval arrest without UV irradiation. Error bars denote the s.e.m. (0.14 MB TIF) [file pgen.1000941.s002.tif]

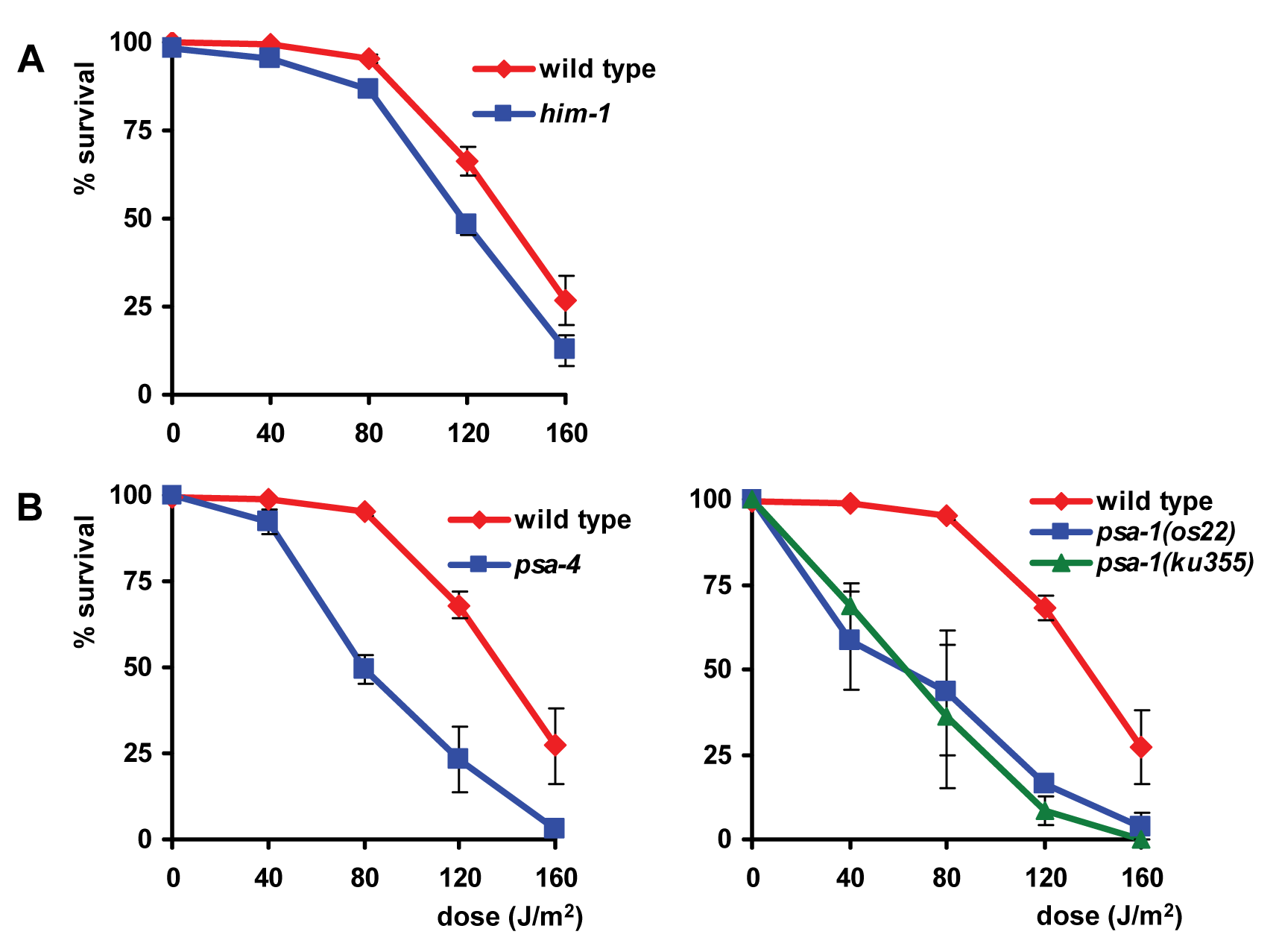

Supplement: Figure S3 — L1 larvae UV sensitivity at 25°C of him-1, psa-4 and psa-1 mutants. UV sensitivity was tested at 25°C. Shown is the L1 larvae UV survival of animals carrying temperature-sensitive mutations in him-1(e879) (A), psa-4(os13) and psa-1(os22 and ku355) (B). Each line represents the mean of at least two independent experiments, each performed in duplicate (typically, n>40). Data for psa-4 and psa-1 were normalized because without UV irradiation these mutants already show some larval arrest. Error bars denote the s.e.m. (0.14 MB TIF) [file pgen.1000941.s003.tif]
