# Supplementary material for: Involvement of Global Genome Repair, Transcription Coupled Repair, and Chromatin Remodeling in UV DNA Damage Response Changes during Development
Source: PLoS Genet. 2010 May 6;6(5):e1000941. doi: 10.1371/journal.pgen.1000941 (PMC2865526; doi:10.1371/journal.pgen.1000941)
Supplement: Table S1 — Genes tested for effect on L1 survival after UV irradiation. Genes tested for their involvement in L1 larvae survival after UV irradiation. If available, mutant alleles were tested. If alleles resulted in lethality or were not available, we applied RNAi to knockdown gene function. Protein domain SNF2 stands for SNF2 family N-terminal domain (Pfam domain PF00176), ARID stands for ARID/BRIGHT DNA binding domain (Pfam domain PF01388). (0.08 MB DOC) [file pgen.1000941.s004.doc]

| **gene** | **protein** | **mammalian** | **RNAi or** |  | **gene** | **protein** | **mammalian** | **RNAi or** |
| --- | --- | --- | --- | --- | --- | --- | --- | --- |
| **name** | **domain** | **ortholog** | **allele** |  | **name** | **domain** | **ortholog** | **allele** |
| *btf-1* | SNF2 |  | RNAi |  | *mep-1* |  |  | RNAi |
| C08B11.3 | ARID |  | RNAi |  | *mrg-1* |  | MRG15 | RNAi |
| C08B11.6 |  | hARPX | RNAi |  | *mys-1* |  | Tip60 | RNAi |
| C16A3.1a |  | SMARCAL1 | RNAi |  | *nurf-1* |  | BPTF | n4295, RNAi |
| C17E4.6 |  | YL-1 | RNAi |  | *pbrm-1* |  | PBRM1 | tm415, RNAi |
| C25F9.5 | SNF2 |  | RNAi |  | *psa-1* |  | SMARCC1 | os22, Ku355 |
| C52B9.8 | SNF2 |  | RNAi |  | *psa-4* |  | SMARCA2 | os13, RNAi |
| *cfi-1* | ARID |  | ky651, RNAi |  | *pyp-1* |  | PPA1/2 | RNAi |
| *chd-3* |  | Mi-2 | eh4, RNAi |  | *rad-26* | SNF2 |  | RNAi |
| *ekl-1* |  | DMAP1 | RNAi |  | *rad-54* |  | RAD54 | RNAi |
| F53H4.6 | SNF2 |  | RNAi |  | *rba-1* |  | CAF1 | RNAi |
| F54E12.2 | SNF2 | TTF2 | RNAi |  | *rbr-2* |  | JARID1A | ok2544, RNAi |
| F59A7.8 | SNF2 | TTF2 | RNAi |  | *ruvb-1* |  | Tip49a | RNAi |
| *flt-1* |  | ACF1 | ok722 RNAi |  | *ruvb-2* |  | Tip49b | RNAi |
| *gfl-1* |  | GAS41 | gk321, RNAi |  | *snfc-5* |  | SMARCB1 | ok622, RNAi |
| H06O01.2 |  | CHD1/2 | RNAi |  | T05A12.4 | SNF2 |  | RNAi |
| H20J04.2 |  | ACF1 | RNAi |  | T23H2.3 | SNF2 |  | RNAi |
| H28O16.2 |  | MCRS1 | RNAi |  | T26A5.8 |  | POLE3 | RNAi |
| *hda-1* |  | HDAC1 | RNAi |  | *tag-192* | SNF2 |  | gk306, gk290 |
| *hda-2* |  | HDAC1 | ok1479, RNAi |  | *tag-246* |  | BAF60a | RNAi |
| *hda-3* |  | HDAC1 | ok1991, RNAi |  | *trr-1* |  | TTRAP | RNAi |
| *isw-1* |  | SMARCA5 | n3294,n3297, RNAi |  | *xnp-1* | SNF2 | XNP/ATR-X | fd-2, tm678 |
| *lact-3* | ARID |  | RNAi |  | Y111B2A.11 |  | EPC1 | RNAi |
| *let-418* |  | Mi-2 | RNAi |  | Y113G7B.14 | SNF2 | TTF2 | RNAi |
| *let-526* | ARID | BAF250 | RNAi |  | Y116A8C.13 | SNF2 |  | RNAi |
| *lin-40* |  | MTA1 | RNAi |  | Y53F4b.3 |  |  | RNAi |
| *lin-53* |  | RbAp48 | n833 |  | Y71H2AM.17 |  | BAF57 | RNAi |
| M03C11.8 | SNF2 |  | RNAi |  |  |  |  |  |
